# *Aspergillus oryzae* as a Cell Factory: Research and Applications in Industrial Production

**DOI:** 10.3390/jof10040248

**Published:** 2024-03-26

**Authors:** Zeao Sun, Yijian Wu, Shihua Long, Sai Feng, Xiao Jia, Yan Hu, Maomao Ma, Jingxin Liu, Bin Zeng

**Affiliations:** 1 College of Chemistry and Chemical Engineering, Jiangxi Science and Technology Normal University, Nanchang 330013, China; sunzeao1010@163.com (Z.S.); a2313066413@163.com (S.F.); 2 College of Pharmacy, Shenzhen Technology University, Shenzhen 518118, China; wuyijian0101@163.com (Y.W.); m18033238251@163.com (S.L.); jx19962021@163.com (X.J.); hbqinghe0806@163.com (Y.H.); ma_maomao@hotmail.com (M.M.)

**Keywords:** *Aspergillus oryzae*, cell factory, industrial enzyme, secondary metabolite

## Abstract

*Aspergillus oryzae*, a biosafe strain widely utilized in bioproduction and fermentation technology, exhibits a robust hydrolytic enzyme secretion system. Therefore, it is frequently employed as a cell factory for industrial enzyme production. Moreover, *A. oryzae* has the ability to synthesize various secondary metabolites, such as kojic acid and L-malic acid. Nevertheless, the complex secretion system and protein expression regulation mechanism of *A. oryzae* pose challenges for expressing numerous heterologous products. By leveraging synthetic biology and novel genetic engineering techniques, *A. oryzae* has emerged as an ideal candidate for constructing cell factories. In this review, we provide an overview of the latest advancements in the application of *A. oryzae*-based cell factories in industrial production. These studies suggest that metabolic engineering and optimization of protein expression regulation are key elements in realizing the widespread industrial application of *A. oryzae* cell factories. It is anticipated that this review will pave the way for more effective approaches and research avenues in the future implementation of *A. oryzae* cell factories in industrial production.

## 1. Introduction

Filamentous fungus, including *A. oryzae*, *Rhizopus oryzae*, etc., widely recognized as a prominent strain in the industrial sector, finds extensive applications across diverse industries, including pharmaceutical manufacturing and food processing [1,2]. *A. oryzae* is an important strain in the industrial application of filamentous fungi, given its long history and vast range of applications. In China and Japan, *A. oryzae* has been extensively employed in the food industry, particularly in the production of traditional fermented foods such as soybean paste and sake. It is also widely recognized as safe (GRAS) by the U.S. Food and Drug Administration (FDA) [3,4]. After an extended period of acclimatization, *A. oryzae* develops a robust system for protein secretion and post-translational modification including glucoamylase, cellulase, and protease [5,6]. Furthermore, *A. oryzae* exhibits robust capabilities in the degradation of various organic matter and plays a pivotal role in the biocycle. In conclusion, *A. oryzae* proves to be an ideal strain for establishing bio-factories that produce industrial enzymes, enabling its significant contribution to the fields of medicine, environment, and food production [7,8,9].

In 2005, Galagan et al. [10] completed the whole genome sequencing of *A. oryzae*, which laid the foundation for genomic research. Such research provides a basis for addressing the challenge of constructing a cell factory from *A. oryzae* using synthetic biological methods. In traditional industry, it exhibits the characteristics of producing multiple hydrolases, such as α-amylases, endo-proteinases, and exo-peptidases. Genome sequence analysis has revealed that *A. oryzae* harbors at least 500 genes with potential practical value in industrial applications [10]. Moreover, the metabolic network of filamentous fungi significantly differs from that of human and yeast cells, facilitating the discovery of novel synthetic routes for secondary metabolites. *A. oryzae* has a clean background of secondary metabolism synthesis and is suitable for producing multiple secondary metabolites, including kojic acid (KA), L-malic acid, and salidroside [11,12,13]. In fact, *A. oryzae* has been found to contain abundant numbers of secondary metabolite gene clusters, surpassing the numbers found in other sequenced species (typically around 5–15) [14,15,16]. This highlights the previously underestimated potential of A. oryzae as an industrial strain.

The main challenge in applying *A. oryzae* is the low level of secondary metabolites and recombinant protein expression in wild-type strains. Traditional methods, such as random mutagenesis, have been applied to increase yields of secondary metabolites in *A. oryzae* [17]. However, these methods suffer from the significant drawback of being inefficient in modifying low-yielding strains. Therefore, it is crucial to establish a rational and efficient breeding methodology based on genetic engineering and metabolic engineering of high-producing strains to enable the commercial application of *A. oryzae*. With the advancement of gene editing technology, the breeding technology of *A. oryzae* has undergone significant development, which is advantageous to developing high-producing cell factories. At present, it is successfully applied to genetic manipulation in *A. oryzae*, including the polyethylene glycol (PEG)-mediated protoplast transformation method, encompassing agrobacterium-mediated transformation, and multiplexed CRISPR/Cas9-mediated genome editing [18,19,20].

**Figure 1:**
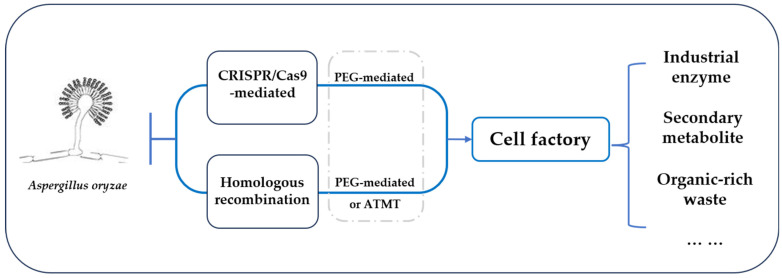
The cell factory based on *A. oryzae* and its applications.

In conclusion, *A. oryzae* demonstrates significant potential for industrial applications and effective gene editing methods have been established. In this review, we aim to highlight recent advances in industrial application of *A. oryzae* research and development, with a particular focus on instances concerning cell factories, which are summarized in Figure 1.

## 2. Cell Factory of Producing Secondary Metabolite by *A. oryzae*

The field of secondary metabolite application in filamentous fungi is rapidly expanding [21]. The gene sequences for multiple *Aspergilli* provide a plethora of predictive information regarding production of various secondary metabolites, ranging from deadly toxins to anti-cancer drugs [22]. The notable advantage of *A. oryzae* is its inability to produce toxic compounds due to dysfunctional or non-expressed genes in the aflatoxin synthesis gene cluster, such as cyclopiazonic acid synthetases and non-ribosomal peptide synthetase [23,24]. In addition, *A. oryzae* possesses a robust metabolic flux that facilitates provision for precursors of polyketides, terpenoids, and peptides [25,26]. Therefore, using *A. oryzae*, we can easily determine the synthetic route for production of beneficial compounds, regardless of whether or not the metabolic is expressed heterologously. In fact, many researchers have already made significant strides in the production of secondary metabolites in *A. oryzae*, as shown in Table 1. These results collectively demonstrate that *A. oryzae* serves as an ideal foundational cell for establishing a secondary metabolite cell factory.

### 2.1. Metabolic Engineering of KA Production in A. oryzae

**Figure 2:**
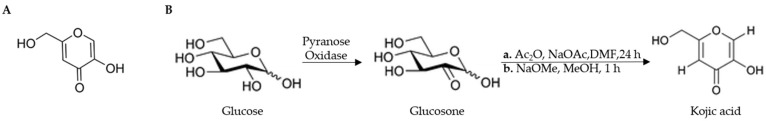
Constitutional formula of KA (**A**); synthetic three-step chemo-enzymatic route (**B**). DFM (dimethyl formamide).

**Figure 3:**
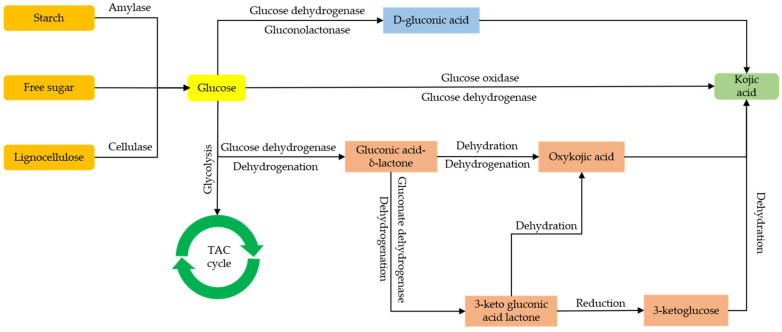
The biosynthetic route of KA production from glucose in *A. oryzae*. Adapted with permission from Refs. [38,40] 2024, Sumit Sharma et al.

KA, a representative secondary metabolite produced by filamentous fungi, finds extensive applications in the cosmetics, pharmaceutical, and food industries. Chemically, it is a pyran-4-one with molecular formula C_6_H_6_O_4_, summarized in Figure 2A [38]. Using a three-step chemo-enzymatic route, Lassfolk et al. realized the preparation of KA from D-glucose via glucosone [39], as depicted in Figure 2B. However, fermentation by filamentous fungi (the producing strains include *A. oryzae*, *Aspergillus niger*) remains the primary method for KA production. The biosynthesis of KA involves complex responses. Numerous researchers have endeavored to identify the metabolic pathway of glucose conversion into KA, with Figure 3 highlighting the main routes that have received significant attention. Firstly, glucose undergoes dehydrogenation catalyzed by GDH, resulting in the formation of gluconic acid δ-lactone. Subsequently, gluconic acid can either undergo dehydrogenation to yield 3-ketogluconic acid lactone or dehydration to produce oxygenated KA. Among these pathways, 3-ketogluconic acid lactone is reduced to 3-ketoglucose, which is further dehydrated to form KA. However, the precise mechanism underlying the conversion of oxygenated KA into KA remains unknown and could be a multi-step process that might be the focus of further investigation [12].

**Figure 4:**
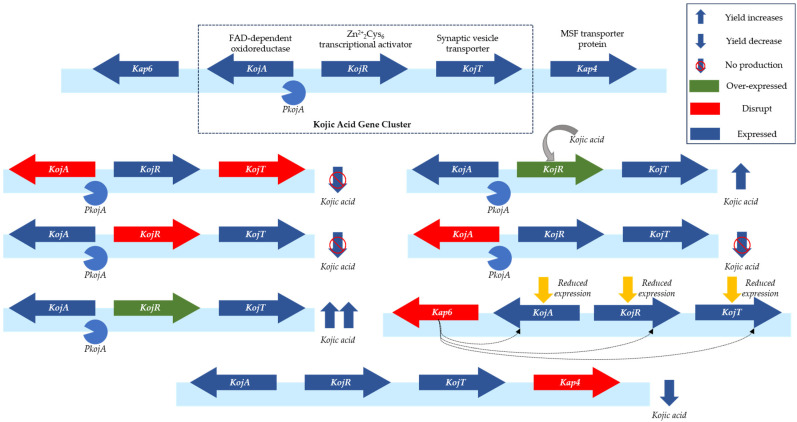
The KA gene cluster in *A. oryzae* and its effect on KA production. Adapted with permission from Refs. [41,42,43,44,45] 2024, Sumit Sharma et al.

Confirmation of the gene cluster as the foundation for constructing an efficient cell factory to produce the secondary metabolites is essential. Previous studies have identified the gene cluster and its associated genes that influence KA production in *A. oryzae*. The gene cluster comprises three genes, namely *kojA*, *kojR*, and *kojT*, which encoding FAD-dependent oxidoreductase, Zn^2+^_2_Cys_6_ transcriptional activator, synaptic vesicle transporter, respectively. Additionally, near the KA gene cluster, we find two genes, *kap4* and *kap6*, that have been found to exert significant influence on KA production. Studies have elucidated their effects on KA production. Knocking out these genes resulted in a deficiency of KA production, whether one or more were knocked out, in *kojA*, *kojR*, and *kojT*, without remedial measure. The research revealed that overexpression of the *kojR* gene within the gene cluster resulted in the highest KA yield, which was 324.28% higher than the control, while exogenous KA impaired the influence of the *kojR* gene [12,41]. Disrupting the *kap4* gene led to a lack of KA production, while disruption of *kap6* repressed KA production, together with the reduced expression of *kojA*, *kojR*, and *kojT*. As shown in Figure 4, these findings suggest that numerous genetic regulations occur during the conversion of metabolic intermediates to direct glucose towards KA synthesis.

Apart from overexpressing the gene cluster, constructing an efficient cell factory also involves the optimal optimization of regulatory elements, such as promoter optimization, overexpression, or disruption of regulatory genes. We reviewed several beneficial studies, which are presented in Table 2. Despite only one gene being modified in these studies, the results demonstrate the relationship between regulatory genes and KA yield, and can be used as a guide for designing a cell factory. Of interest, autophagy processes, particularly autophagy of the nucleus and protein targeting to the vacuole, can influence secondary metabolite production in filamentous fungi [46]. Additionally, another study revealed that *AoZip2* genes from the ZRT/IRT-like protein (ZIP) family also affect kojic acid expression, as kojic acid expression was downregulated when the *AoZip2* gene was overexpressed [47]. This finding might be attributed to the responses to metal ions in kojic acid production in *A. oryzae*. These results provide valuable insights into building an efficient cell factory, where the overexpression and disruption of a series of genes are required to increase KA yield as part of system engineering.

### 2.2. Molecular Mechanism for Secondary Metabolite Secretion in A. oryzae

In *A. oryzae*, several secondary metabolites possess unique molecular mechanisms of secretion. However, the transportation processes involved in many of these metabolites remain unclear. Regarding the secretion of citric acid in filamentous fungi, extensive research has been conducted [53]. Initially, *CtpA* and *YhmA* play a crucial role in transporting citric acid from the mitochondria to the cytoplasm, and their transporters are localized to the mitochondrial membrane [54]. Subsequently, a dedicated transporter facilitates the transportation of citric acid from the cytoplasm to extracellular secretion. The transcription of *cexA*, the gene encoding this transporter, is regulated by *LaeA* [55]. It has been observed that overexpression of the *cexA* gene in *A. oryzae* cells enhances citric acid secretion, suggesting that *cexA* is a limiting factor for this process [56]. These findings highlight the importance of optimizing the secretion mechanism in *A. oryzae* to increase secondary metabolite yield.

## 3. Cell Factory for Producing Industrial Enzymes with *A. oryzae*

Enzymes offer several advantages in green production, including milder operating conditions, enhanced product specificity, efficient resource utilization, and pollution reduction [57]. As green production becomes more prevalent, the global industrial enzymes market is expanding at a rapid pace, estimated to be valued at USD 7.42 billion in 2023 [58]. Demand for these products in the animal feed, pharmaceutical, and nutraceutical sectors is expected to experience significant growth, driven by the expansion of meat production and the pharmaceutical industry. In 2023, the microorganisms segment dominated the industrial enzyme market, accounting for the highest share at 85.49%, primarily due to their low production cost and easy availability. Among the various characteristics of *A. oryzae*, as a production strain for fermented food, one of the most important is its ability to produce significant amounts of extracellular hydrolytic enzymes, such as amylolytic and proteolytic enzymes [59,60]. Furthermore, compared with *Escherichia coli* and *Saccharomyces cerevisiae*, *A. oryzae* is a superior host for expressing proteins with intricate structures, owing to its stronger posttranslational modification function. Therefore, *A. oryzae* is well suited for the production of industrial enzymes. Table 3 lists many various relevant studies related to production of industrial enzymes in *A. oryzae*. In general, *A. oryzae* has a wide range of applications for industrial enzyme production. However, due to its inefficiency, heterogeneous expression of proteins in *A. oryzae* is a challenging event, which may be related to complicated secretory pathways.

### 3.1. α-Amylase Production in A. oryzae

Amylases, which were first identified in the eighteenth century and are found in bacteria, fungi, animals, and plants, are among the enzymes initially used in industrial production [76]. Microbial-based commercial production of amylases accounts for approximately 30% of the global enzyme market [77]. α-amylase is a prominent secretory protein in *A. oryzae* and finds extensive application in industrial enzymes [78]. Understanding of its regulatory mechanisms and secretory pathway is the key to increasing its production.

The expression of α-amylase genes in *A. oryzae* is induced by starch and malto-oligosaccharides. The gene-inducible expression is regulated by *AmyR*, one of the fungal-specific Zn(II)_2_Cys_6_-type transcription factors [78]. The *AmyR* gene is usually constitutively expressed and localizes in the cytoplasm. However, upon addition of isomaltose to the medium, *AmyR* is rapidly transferred into the nucleus [79]. In contrast to *A. nidulans*, c-terminal truncation of *AmyR* in *A. oryzae* leads to the loss of its function, indicating species-specific differences in *AmyR* among *Aspergillus* species. In multicellular organisms, the regulation of gene expression involves key factors known as morphogens that play a role in organizing gene expression [80]. However, filamentous fungal cells are highly polarized, and generally the nucleus is at some distance from the tip of the hypha, which distinguishes *A. oryzae* from others [81,82]. Furthermore, the regulation of the cell cycle in *Aspergillus* species is synchronized, which differs from most multicellular systems [83]. Consequently, the mechanism of *AmyR* activation in *A. oryzae* is more complex. Maltose is incorporated by the maltose permease *MalP* and converted to isomaltose by the transglycosylation activity of the intracellular α-glucosidase *MalT* [84]. This mechanism may have a beneficial effect on increasing amylase production. The α-amylase (*amyB*) gene promoter also is commonly used for high-level expression of heterologous genes in *A. oryzae* [59].

### 3.2. Molecular Mechanism for Protein Secretion in A. oryzae

In the *A. oryzae* genome, there are 135 genes predicted as secretory protease by signal peptide, including amylase genes [85]. Solid-state culture is a commonly used industrial method to cultivate *A. oryzae* cells to produce industrial enzymes, as secretory proteins are produced to a greater extent in solid-state culture compared with submerged culture [86]. However, there are certain proteins that are not secreted in solid-state culture, unlike submerged culture, such as the glucoamylase-encoding gene *glaB* [87]. These findings suggest that there is a molecular mechanism governing protein secretion in *A. oryzae*.

**Figure 5:**
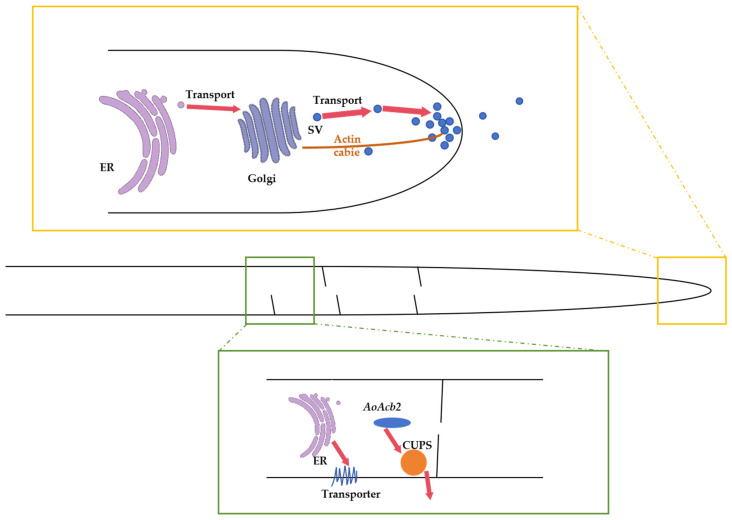
Protein secretion pathways in *A. oryzae* filaments. SV, secretory vesicle; CUPS, compartment for unconventional protein secretion.

The secreted protein contains a signal peptide at the N-terminus, which initially targets it to the endoplasmic reticulum (ER). It is then transported from the ER to the plasma membrane via vesicles through the Golgi apparatus before finally being secreted outside of the cell. During its passage through the ER and Golgi, secreted proteins undergo modifications through the addition of N- and/or O-glycan chains, which serve functions such as protein stabilization and localization. The mechanism of N-glycosylation is highly conserved in filamentous fungi. Within the ER lumen, secreted proteins undergo the calnexin/calreticulin cycle prior to transport to the Golgi. The Glc_3_Man_9_GlcNA_2_ moeity is attached to the Asn residue of the glycoprotein, which is then further processed by glucosidases I and II to remove the Glc moiety [88]. The remaining individual GlcNAc moiety on the secreted expressed protein is important for maintaining protein structure and function, and it affects enzyme activity [89]. In *A. oryzae*, N-glcNAc-modified proteins are produced extracellularly through the expression of endo-β-N-acetylglucosaminidase (ENGase) located on the Golgi membrane [90]. In addition, the fluorescence localization signals show that secreted expressed proteins are mainly secreted from the hyphal tip in *A. oryzae* [91]. In this process, the actin and microtubule cytoskeletons are indispensable. There is septum-directed secretion in *A. oryzae* [92]. This process is illustrated in Figure 5.

## 4. Cell Factory of Utilized Organic-Rich Waste by *A. oryzae*

**Figure 6:**
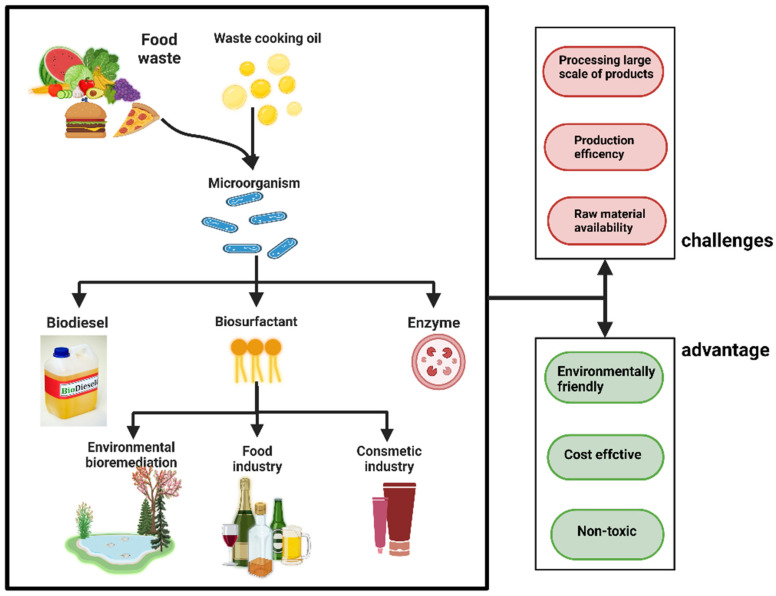
Application prospects, and advantages and disadvantages of organic-rich waste treatment by cell factory.

In modern society, a significant amount of organic-rich waste is generated, posing harmful effects on the environment. The green production standard advocates for the appropriate treatment and recycling of organic-rich waste [93]. Utilizing cell factories to produce valuable commodities based on this waste is an effective method of waste treatment [94,95]. Current research suggests that the primary products generated by cell factories include biosurfactants, enzyme preparations, single-cell proteins, and polyols [96,97,98]. Additionally, certain types of organic-rich waste can be utilized as a suitable growth medium for specialized strains (including genetically modified and naturally screened strains), thereby inducing the synthesis of relevant secondary metabolites. This use of organic-rich waste is powerful for sustainable development of the circular bioeconomy. In Figure 6, we summarize some possible products and the strengths and weaknesses of the cell factory to treat organic-rich waste. The primary challenge currently faced by cell factory relates to identifying microbial strains that exhibit a remarkable capacity for efficient utilization of organic-rich waste, as well as implementing effective metabolic engineering strategies to optimize performance. *A. oryzae* possesses an efficient hydrolase system, which includes phytases, β-glucosidases, and other enzymes [99,100,101,102]. Furthermore, *A. oryzae* is an ideal candidate for constructing a cell factory due to its ability to withstand high osmolality and other challenging environments. Extensive research in this field has yielded numerous intriguing and valuable discoveries, highlighting the significance and potential for further exploration of *A. oryzae*. 

### 4.1. Cell Factories for Processing Food Waste

**Figure 7:**
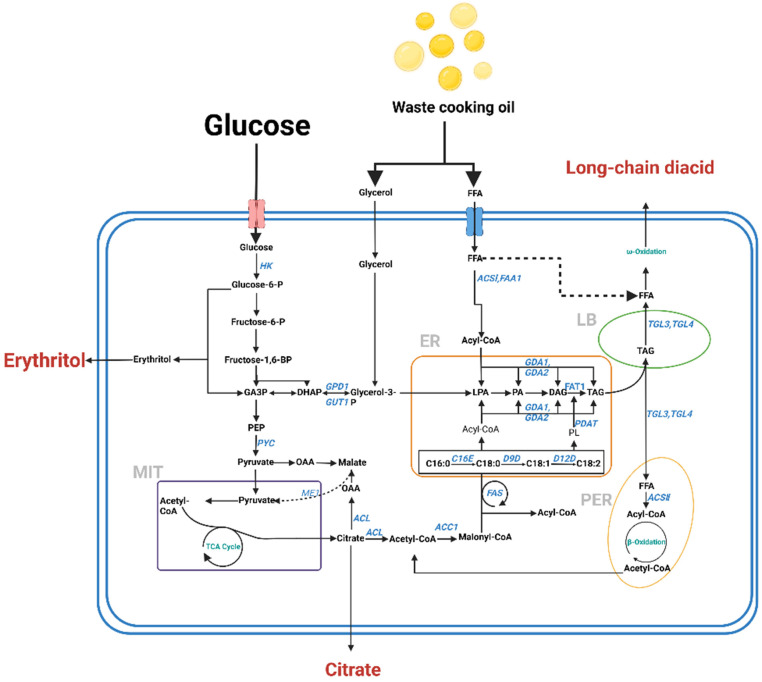
Microbial metabolism pathways involved in waste cooking oil degradation. Abbreviations: MIT (mitochondria), TCA cycle (tricarboxylic-acid cycle), LB (lipid body), DHAP (dihydroxyacetone phosphate), GA3P (glycerol-3-phosphate), PEP (phosphoenolpyruvic acid), OAA (oxaloacetic acid), PA (phosphatidic acid), LPA (lysophosphatidic acid), DAG (diacylglycerol), TAGs (triacylglycerols), FFA (free fatty acids), PYC (pyruvate carboxylase), ME (malic enzyme), ACL (ATP-citrate lyase), ACC (acyl-CoA carboxylase), FAS (fatty acid synthase), GUT1 (glycerol kinase), GPD1 (NAD^+^ dependent G3P dehydrogenase), GUT2 (FAD^+^ dependent G3P dehydrogenase), SCT1 (G3P acyltransferase), SLC1 (LPA acyltransferase), PAP (PA phosphohydrolase), DGA1 and DGA2 (DAG acyltransferases I and II), TGL4 (TAG intracellular lipase), TGL3 (a positive regulator of TGL4).

The food processing industry represents one of the primary sources of organic-rich waste [103]. The composition of food waste is complex, which makes it difficult to manage. Waste cooking oil (WCO) poses a challenge in food waste treatment due to its toxic effects on certain microorganisms [104]. Figure 7 illustrates the principal metabolic pathways employed in a commonly used model cell factory for studying the degradation of WCO. The metabolic pathway of WCO within an organism is closely linked with intracellular lipid metabolism and effectively bypasses the tricarboxylic acid cycle. For instance, Hui Huang et al. [105] demonstrated the beneficial effects of the thiolytic enzyme gene in the utilization of WCO, as observed during the study of ergosterol. Another study utilized *A. oryzae* to produce single cell protein from waste-derived volatile fatty acids (VFAs) and achieved a biomass yield of 0.26 g dry biomass/g VFAs_fed_ [106]. Furthermore, the presence of cooking oil exhibited a significant influence on biomass growth. Muhammad Tahir Nazir et al. analyzed the biomass obtained from *A. oryzae* for protein, fat, and alkali-insoluble material, revealing a biomass growth of 16 g/L with the addition of oil compared with 4 g/L without oil [107].

In addition to WCO, other wastes generated during food production can be digested by *A. oryzae*. Natsumi Iwamoto et al. found that abalone viscera fermented by *A. oryzae* 001 had an inhibitory effect on blood pressure elevation, possibly due to the isolation of L-m-tyrosine, a unique substance in fermented abalone viscera, which was identified as a single ACE-inhibitory amino acid for the first time [108]. Brewer’s spent grain (BSG) is the main solid by-product of the brewing sector. Research has shown that submerged cultivation of BSG with *A. oryzae* can significantly enhance the protein content, with the highest increase observed at 34.6% (from 22.6%), and a concurrent decrease in the content of polysaccharides by up to approximately 50% [109]. Barley bran (BB) is a by-product of the milling process. Solid substrate fermentation (SSF) of BB was performed with *A. oryzae* for 7 days, resulting in an improvement in the bioactive compounds of BB, including increased levels of ascorbic acid (107.15 µg/g), gallic acid (405.5 µg/g), catechin (88.3 µg/g), vanillin (40.89 µg/g), and resorcinol (20.7 µg/g) [110]. Furthermore, Ikram-Ul-Haq et al., using a soya bean meal medium, conducted submerged fermentation with *A. oryzae* to produce β-galactosidase, with a maximum productivity of 112.34 ± 0.23 U/mL/min [111]. In general, utilizing food waste to derive bioactive molecules through *A. oryzae* is a practical approach.

Moreover, intensive research has demonstrated that food waste can be utilized for production of biofuels, such as ethanol and lipids for biodiesel. *A. oryzae*, the strain used for sake production, is an ideal candidate for constructing a cell factory. Joanna Kawarygielska et al. reported *A. oryzae* final product yields ranging from 0.29 to 0.32 g EtOH/g and 0.20 to 0.22 g biomass/g bread waste, on the second fermentation [112]. Abdullah Bilal Ozturk et al. conducted experiments to test the production of bio-butanol through fermentation of Japanese steamed rice using *A. oryzae* and *Clostridium acetobutylicum*, and the output was (10.91 ± 0.16) g/L [113].

### 4.2. Cell Factory for Processing Agricultural Waste

Lignocellulose constitutes the primary component of waste generated in agricultural production, such as corn cobs, straw chaff, etc. [114]. Additionally, lignocellulosic biomass serves as a crucial raw material for extracting bio-based fuels and other value-added products, including organic acids, fructans, phenols, mono-pentose/oligosaccharides, and hexose [115,116,117]. Microbial enzymatic saccharification of lignocellulose represents an effective approach for sustainable utilization of this resource [118,119]. *A. oryzae*, with its extracellular cellulase activity, emerges as an ideal strain for constructing a cell factory to process agricultural waste [94,120].

Alberto Robazza et al. utilized pyrolysis waste derived from lignocellulose as a culture substrate for L-malic acid production through inoculation of *A. oryzae*, achieving yields of up to 0.17 mM/mM [121]. Apart from malic acid, organic acids found in plants are also popular regenerative products. Ignacio Cabezudo et al. employed *A. oryzae* for gallic acid production, utilizing soybean hull and grape pomace as supporting substrates, resulting in the production of 0.36 g of gallic acid per gram of tannic acid and 7.2 g/L of fermentation medium after 72 h of incubation [69]. Moreover, lignocellulosic biomass treated with *A. oryzae* is commonly used in animal feed. A study was conducted to valorize this agricultural waste into alternative ruminant feed using exogenous fibrolytic enzymes (EFE) through fermentation of a mixed culture of Aspergillus strains [122].

## 5. Discussion and Conclusions

The global market demand for biological resources, such as secondary metabolites and industrial enzymes, continues to increase with the expansion of the pharmaceutical and healthcare markets. To address this, the use of cell factories built through synthetic biology has emerged as an effective solution for achieving green production of these products. As an organism with biosafety characteristics, *A. oryzae* possesses abundant gene resources for secondary metabolite synthesis and an efficient protease expression system, making it an ideal chassis organism for constructing cell factories. Research on utilizing *A. oryzae* to construct cell factories for industrial product production is gaining momentum.

Metabolic engineering strategies and synthetic biology tools have the potential to significantly enhance the performance of *A. oryzae*, encompassing synthesis capacity, growth performance, and stress resilience [123,124]. Despite the sequencing of the genomic information of *A. oryzae*, the metabolic pathways of numerous secondary metabolites remain elusive, presenting a major challenge in related research. Furthermore, the secretion pathways of secondary metabolites in *A. oryzae* have not been extensively characterized, thereby limiting the production of these metabolites. Constructing cell factories can be a promising approach to address these challenges.

Considering its exceptional protein secretion system and post-translational modification pathway, *A. oryzae* is considered a promising candidate for a protein cell factory [125]. Up to now, our research has focused on investigating the secretory expression system of proteins in *A. oryzae*, leading to successful high expression of numerous homologous proteins [126]. Nevertheless, limited knowledge about the regulation of heterologous protein expression in *A. oryzae* and the relatively low efficiency of such expression currently hinder its industrial implementation [127]. With advancements in proteomics and the utilization of novel gene editing technologies in *A. oryzae*, we are optimistic about achieving efficient expression of heterologous proteins in the *A. oryzae* cell factory [19,128].

## 6. Expectations

As a biosafe strain, *A. oryzae* possesses a highly efficient secondary metabolite synthesis pathway and protein secretion expression system. It has found extensive utilization in traditional industries, particularly in food production. Moreover, through the integration of proteomics and genetic engineering techniques, *A. oryzae* has emerged as an optimal candidate for constructing cell factories. Therefore, to facilitate the wider industrial application of the *A. oryzae* cell factory, comprehensive studies on its secretion system and protein expression regulation mechanism are of utmost importance.

## Figures and Tables

**Table 1:** Secondary metabolites produced by *A. oryzae*.

Metabolite	Gene Source	Substrate	Strain	Yield	Reference
2,4′-dihydroxy-3′-methoxypropiophenone	Congeneric	Sucrose	RIB40	-	[27]
Pyrones	Congeneric	Solid rice medium	- ^1^	-	[28]
N, N-dimethyldecylamine N-oxide	Congeneric	Potato dextrose agar	MK674278	-	[29]
Orsellinic acid	Heterologous	Maltose	NSAR1	340.41 mg/Kg	[30]
Non-ribosomal peptide pigments	Congeneric	Glucose	BCC 7051	265.09 ± 14.74 mg/L·d	[24]
Flavonoid aglycones and cajaninstilbene acid	Congeneric	Cajanus cajan cell suspension cultures	Y-29	-	[31]
L-malic acid	Congeneric	Acetate	DSM 1863	12.08 ± 1.25 g/L	[32]
L-malic acid	Congeneric	Glucose	DSM 1863	178 g/L	[33]
L-malic acid	Congeneric	Glucose	DSM 1863	-	[34]
L-malic acid	Congeneric	Glucose	FMME-S-38	164.9 g/L	[35]
KA	Congeneric	Glucose	-	139.24 g/L	[36]
Glucosamine	Congeneric	Potato dextrose broth	NCH-42	0.31 g/g	[37]

^1^ The uncertain strain was screened from the environment by researchers.

**Table 2:** Several regulatory genes and their effects on KA yield.

Strain	Gene Name	Encoded Protein	Acting Site	Genetic Manipulation	KA Yield	Reference
3.042	*kojR*	Zn^2+^_2_Cys_6_ transcriptional activator	-	Overexpressed	32.5 g/L	[12]
RB40	*hirA*	Histone chaperon	Transcription	-	-	[48]
3.042	*Aokap2*	Cell surface ferric reductase	*laeA* & *kojA*	Overexpressed	Increased	[49]
3.042	*Aokap5*	C2H2-type zinc-finger protein	kojT promotor	Overexpressed	Increased	[50]
3.042	*Aokap1*	Kojic acid related protein 1	*kojA*, *kojR* and *kojT*	Disrupted	Increased	[51]
3.042	*AozfA*	Zinc finger protein	Transcriptional activator	Overexpressed	Reduced	[52]
RB40	*Aoatg8*	Enables phosphatidylethanolamine binding activity and protein tag	Autophagy	Disrupted	Increased	[46]
RB40	*AoZip2*	IRT-like protein	Response of metal ions	Overexpressed	Reduced	[47]

**Table 3:** Producing industrial enzymes in *A. oryzae*.

Strain	Enzyme	Source	Position	Acetive	Reference
MN894021	Xylanase	Homologous	Extracellular	0.37 U/mL	[61]
ATCC 10124	Xylanase	Homologous	Extracellular	11.90 U/g DS ^1^	[62]
SBS50	Phytase	Homologous	Extracellular	506.12 U/g	[63]
BM-DIA	Fructosyltransferase	Homologous	Extracellular	1.59 U/mL	[64]
-	α-amylase	Homologous	Extracellular	9868.12 U/gds	[65]
NRRL695	α-amylase	Homologous	Extracellular	14.076 U/mL	[66]
S719	β-fructofuranosidase	Homologous	Extracellular	155.4 U/mL	[67]
S719	Fructosyltransferase	Homologous	Extracellular	12 U/mL	[68]
NRRL695	Tannase	Homologous	Intracellular	-	[69]
NRRL1560	1,4-α-D-glucan glucohydrolase	Homologous	Extracellular	-	[70]
DRDFS13	Milk-clotting protease	Homologous	Extracellular	137.58 U/mL	[71]
ISL-9	Pectin lyase	Homologous	Extracellular	9.26 U/mL	[72]
HML366	Endoglucanase	Homologous	Extracellular	-	[73]
AOK11	Recombinant tannase	Heterogenous	Extracellular	-	[74]
NSPlD1	Polyketide synthase	Heterogenous	Intracellular	-	[75]

^1^ DS, dried solids.

## Data Availability

No new data were created or analyzed in this study. Data sharing is not applicable to this article.
